# Reconciling the Chicago River for birds and people

**DOI:** 10.1371/journal.pone.0256733

**Published:** 2021-09-02

**Authors:** Alexis Dyan Smith

**Affiliations:** Biological Sciences, University of Illinois at Chicago, Chicago, IL, United States of America; Feroze Gandhi Degree College, INDIA

## Abstract

The Chicago River’s north branch intersects multiple urban land uses, including residential, industrial, commercial, and recreational. The north branch also supports a diversity of birds exploiting a variety of resources and structures along the river as habitat. From three breeding seasons of point count surveys, I assess the breeding bird communities in four different sections, representing four different restoration or management styles. These four river sections are also very different with regards to the surrounding neighborhood demographics. These data serve as both a baseline for future studies to evaluate restoration projects along the Chicago River, and as a snapshot to compare bird diversity and community composition between these river sections given current conditions. Unsurprisingly, the section of the river with the most extensive and longest established restoration effort had the highest species richness (number of species) of native birds. In terms of aquatic and riparian birds, however, that section was comparable to river sections with much less management in measures of both species richness and species composition. I discuss ways that river restoration efforts can be sensitive to demographic context, to avoid contributing to eco-gentrification and displacement.

## Introduction

Urban rivers are valuable as habitat and as spaces for human recreation [1], but they are altered in multiple ways that affect their function and habitat quality when compared to rivers in less human-dominated areas. The increased impervious surface in urban areas alters a river’s flow and can lead to higher levels of pollutants [2,3]. Anthropogenic obstructions can restrict aquatic species dispersal [4,5]. This reduced connectivity leads to changes in aquatic communities, particularly to a decrease in species richness (number of species) with an increase in human density [6].

Because of their ability to fly, birds are less affected by issues of connectivity than other taxa. However, other aspects of urbanization affect river-associated birds. River channelization often increases the speed of the river flow, which can impact species with specific flow requirements [7]. River channelization also reduces the area of river edge habitats and vegetation. Riparian vegetation is important not only for nutrient cycling and temperature regulation, it is also responsible for creation of woody debris [8]. Woody debris is often intentionally removed from urban rivers, but it creates important habitat for multiple taxa, and birds in particular [9].

In addition to improving habitat quality for multiple taxa, river restoration can support economic revitalization [8], but this economic revitalization can have negative consequences for economically vulnerable residents. Literature on eco-gentrification offers multiple examples of urban greening or urban restoration efforts raising rents and displacing residents [10]. Restoration targets have shifted from historic, natural references to those more focused on human needs and benefits [11]. Targeted human needs and benefits typically include things like recreation, public health, and economic development. Assuredly, housing is a human need at least as much as is recreation, but the needs and benefits of economically vulnerable residents are rarely prioritized in urban greening efforts.

The original motivation for this study was to establish baseline data on the breeding birds of the Chicago River’s north branch. I had learned about floating gardens being constructed by the group Urban Rivers (www.urbanriv.org) across the river from Goose Island. Baseline data would make it possible to evaluate the effect that this project might have on breeding birds in the future. I collected data not only where this project was planned to take place but also at other points along the north branch to serve as comparison. The other study points were selected without substantial prior knowledge of these river habitats.

Purely by chance however, at each grouping of point counts I encountered other river restoration or urban greening projects, either planned, in progress, or established. These projects and the surrounding neighborhoods are all qualitatively very different from each other, providing an interesting cross-section of restoration strategies and human demographics. This experience led to contemplation about river restoration in human-dominated landscapes, with a particular focus on meeting the needs of a diversity of humans as well as a diversity of birds. While this study still presents a baseline to which future researchers can compare when studying birds along the Chicago River, comparing between groups within this present snapshot is also informative. Understanding urban biodiversity conservation within the context of the people who share the city is useful to urban planners and conservationists alike, who may struggle to reconcile the needs of wildlife with the needs of humans.

Here I describe the breeding bird communities along a very urban part of the Chicago River’s north branch. In four different river sections, representing four different restoration or management styles influenced by land use and demographics, I compare the species assemblages within and between these sections, to understand how these different management styles may influence beta diversity throughout the study area. In addition to management, bird diversity could also be influenced by local and landscape factors such as canopy cover, population density, or proximity to a major road. I weigh the relative importance of these variables in explaining bird species richness, particularly aquatic-associated bird species richness. I discuss the results within a social context as well as a conservation context.

## Methods

### Study area

The city of Chicago, Cook county, IL (USA; 41.8781°N, 87.6298°W) is situated on the coast of Lake Michigan, on land of the Three Fires Confederacy (Potawatomi, Odawa, and Ojibwe Nations) and other Tribal Nations in the Great Lakes Region. Home to more than 2.7 million people, Chicago is the third most populous city in the United States. Chicago has a humid continental climate, marked by hot summers and cold winters.

The Chicago River has undergone substantial changes since the city was granted its first town charter in 1835. Notably, the direction of flow of much of the river was reversed in 1900 with the opening of the Chicago Sanitary and Ship Canal [12]. Various stretches of the river were host to the booming meat-packing and other industries in the late 19^th^ and early 20^th^ centuries, and legacy pollutants remain [13]. Wetland area in Cook County declined by 40% over the 20^th^ century [14], and changes to the Chicago River contributed to this loss. Where the edges of the river once sloped gently into shallow marshes, there are now steep sides of corrugated metal, concrete, or other human-made materials.

**Fig 1:**
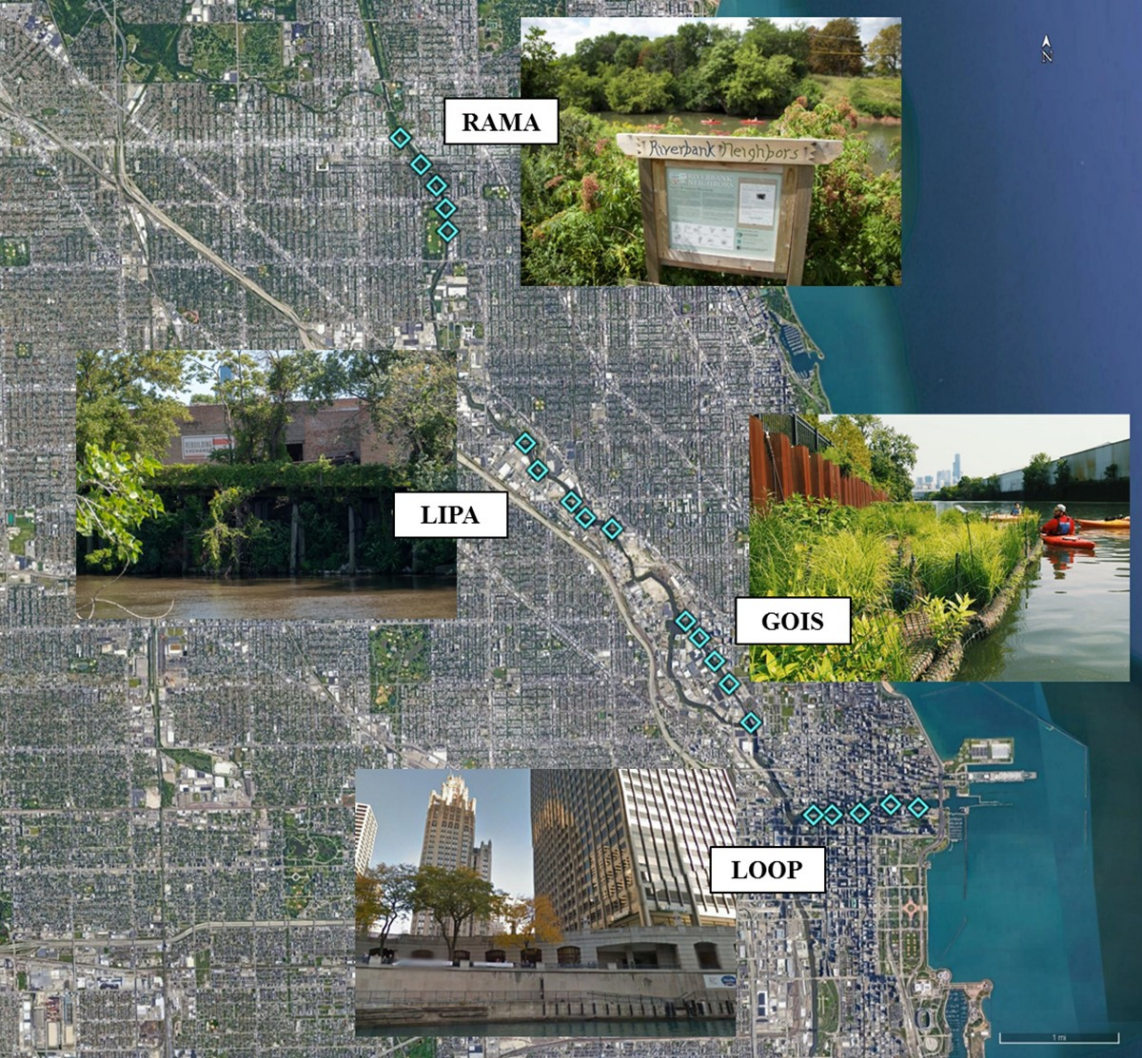
Overview of study sites. Aerial view of study points along the Chicago River, with characteristic imagery from each of the four study river sections. Aerial imagery is from Google Earth Pro (imagery date 5/4/2019). Inset images are from (from top to bottom) Riverbank Neighbors Park, Joy Elaine Davenport, Urban Rivers, and Google Earth StreetView.

I selected 20 study points in the river’s north branch and outlet to Lake Michigan, five per each of the four river sections described below (Fig 1). The study points were each at least 250 m apart as recommended by Ralph et al. [15], and each study river section was at least 1 km from the next study river section.

#### Albany Park/Ravenswood Manor/Horner Park (RAMA)

This primarily residential section of the river is bordered by Ronan Park on the north end and Horner Park on the south end. Both of these parks have areas managed for wildlife, such as Ronan Park’s Bird & Butterfly Sanctuary and Horner Park’s river shoreline restoration, which began in 2013. Additionally, neighborhood residents established the Riverbank Neighbors Park, which features native plants and a more natural shoreline, across the river from Horner Park.

#### Lathrop Homes/Lincoln Park (LIPA)

Historic Lathrop Homes, one of Chicago’s first public housing developments, sits just north of this section of river. This section of the river is characterized by big box stores with large parking lots. Most of this section of the river has not been intentionally managed for wildlife and biodiversity, but a number of primary succession plant species such as white mulberry (*Morus alba*) have become established, softening the hard edges of the river. At the northernmost study point of this section, the Diversey Floating Wetland was created but not maintained, and I observed trash covering the artificial wetland structure at every visit. After this study concluded, the Lathrop Riverwalk finished construction, coinciding with the controversial redevelopment of Lathrop Homes into mixed-income housing. The riverwalk was built with what seems to be hostile architecture to exclude unhoused people. At other points along this river section, unhoused people live in tents under the bridges.

#### Goose Island (GOIS)

On the east side of the river from Goose Island, there are floating gardens that will become part of Urban Rivers’ “Wild Mile,” currently in progress. The gardens feature native plants with signs identifying the species, which is in line with the project’s (www.wildmilechicago.org) stated goal of creating “a new environment for 1) habitat, 2) recreation, and 3) education.” At the time of this study, the completed floating gardens were installed along a short riverwalk behind a chain health food store. Although the riverwalk is not yet continuous along the entire length of this section of river, most of this river section is accessible to pedestrians.

#### The Loop/New East Side (LOOP)

This section of river is the most densely urban and also the most accessible to pedestrians of the study area. One can walk continuously along the Chicago Riverwalk from the Franklin-Orleans Street Bridge to the entrance of Lake Michigan. The Chicago Riverwalk is a major attraction for Chicago tourists as well as residents. There are restaurants, bars, cafés, boat tours, and more along the riverwalk, and one can commonly see joggers and dog-walkers there.

### Point counts

According to recommendations by Ralph et al. [15] and Dobkin and Rich [16], I conducted fixed-radius point counts at each of the 20 points along the river. At each point, I recorded the species and abundances of all birds seen and heard within 100 m for 10 minutes. Bird species were typically identified by songs or calls, but often by sight. I visited each point two times during each breeding season for three years. The first round of point counts at each site was conducted during the last week of June, and the second round during the first week of July in the years 2017 through 2019. All point counts were conducted by the same observer, between 7:30 AM and 10:00 AM on days without rain or excessive wind.

### Geographic information systems

Study points were selected in Google Earth Pro with no prior knowledge of the sites on the ground. A few of the points needed to be adjusted slightly on site when it was discovered that they were not accessible by foot. Although the point counts were conducted on land on the edges of the river, I created a shapefile with the study points in the middle of the river nearest to the actual location. This shapefile was created by making placemarks in Google Earth and then converting to ArcGIS using the KML to Layer tool.

The four explanatory variables (canopy cover within 50 m and 1 km, population density, and distance to the nearest primary or secondary road) were all calculated in ArcGIS version 10.7.1. For calculating canopy cover, I created a composite tool using ArcGIS model builder. This composite tool uses a series of standard ArcGIS tools to derive the percent of canopy cover from a raster file of land cover. I used the high-resolution Cook county 2010 land cover file from CMAP Data Hub. The composite tool clips the raster file to the desired radius from each point in the shape file of interest, reclassifies the cells in the clipped raster to 1 for tree canopy and 0 for all other land cover categories, then uses the Focal Statistics (mean) and Sample tools to extract the proportion of canopy cover within the desired radius of each study point. I chose a radius of 50 m to approximate the amount of shade directly over the river and locally available vegetation and woody debris, and a radius of 1 km to account for landscape level effects of canopy cover.

I calculated distance to a major (primary or secondary) road using the Near tool and 2017 TIGER/Line data for Cook county. Population density was approximated from 2010 US Census data at the block level. I included all blocks for which its centroid fell within a 500 m buffer, and divided the population total by the total area of the included blocks.

### Statistical analyses

I combined all observations of birds over all three years at each point, and then removed introduced species from these counts, for a single measure of native bird species richness (number of species) for each point. I narrowed this group further for a measure of “aquatic” bird species richness. Most of the taxa included in this group were included for obvious reasons, such as herons, ducks, and gulls. However there are a few species that I included that live in a diversity of habitats but that are either very active foragers in riparian habitats or have been shown to prefer habitat near water. I compared the river sections to each other in terms of all native bird species richness and aquatic bird species richness using the Wilcoxon test [17].

To determine which of the four explanatory variables best explain species richness of all native birds and of aquatic birds in the study area, I compared linear regression models using Aikake’s information criterion [18] with an adjustment for small sample sizes (AICc). I used simple linear models rather than generalized linear models because both of the response variables (species richness of all native birds and species richness of aquatic birds) were approximately normally distributed. Additionally, I used the Shapiro–Wilk test to test for normality of regression residuals for all linear models. For all models, the sample size was 20 study points. For each of the response variables, I also ran a null model. Models that outperformed the null model were identified by a lower AICc value. AICc analyses were conducted using the package “AICcmodavg” in R [19].

I analyzed aquatic bird community beta diversity in two ways: an analysis of similarity (ANOSIM) and a dendrogram. Using Whittaker’s beta diversity metric, I constructed a matrix of pair-wise beta diversity values between the aquatic bird communities in the 20 sample points. This matrix was the basis for both the ANOSIM and the dendrogram. An ANOSIM compares the similarity of the communities within each section to the similarity of all the communities across the entire study area. A dendrogram is a way to visualize the beta diversity across the study area; communities with more similar species composition will be clustered more closely together. All community analyses were conducted using the package “vegan” in R [20].

## Results

A total of 42 bird species were detected across all sites and across all three years, 39 of which were native species and 18 of which were classified as “aquatic” native species. Ring-billed gulls (*Larus delawarensis*) were encountered at all 20 points. Nearly as widespread were Caspian terns (*Hydroprogne caspia*; 19 points), and barn swallows (*Hirundo rustica*; 17 points). Median native bird species richness at a point was 14.5 species (min. = 2 species, max. = 23 species). Median aquatic bird species richness at a point was 8 species (min. = 2 species, max. = 13). The birds classified as aquatic and the locations where they were found are listed in Table 1.

**Table 1:** Species names and locations where detected for all aquatic birds.

Species	GOIS					LIPA					LOOP					RAMA				
	1	2	3	4	5	1	2	3	4	5	1	2	3	4	5	1	2	3	4	5
**American Coot (*Fulica americana*)**		●																		
**Barn Swallow (*Hirundo rustica*)**	●	●	●	●	●	●	●	●	●	●	●				●	●	●	●	●	●
**Black-crowned Night-Heron (*Nycticorax nycticorax*)**		●	●	●		●	●	●	●		●					●	●	●	●	●
**Blue-winged Teal (*Spatula discors*)**		●	●																	
**Canada Goose (*Branta canadensis*)**	●	●	●			●	●	●	●	●						●	●	●		
**Caspian Tern (*Hydroprogne caspia*)**	●	●	●	●	●	●	●	●	●	●	●	●	●	●	●	●		●	●	●
**Common Grackle (*Quiscalus quiscula*)**			●	●		●		●	●	●						●			●	●
**Double-crested Cormorant (*Phalacrocorax auritus*)**								●		●						●				●
**Eastern Kingbird (*Tyrannus tyrannus*)**																			●	
**Great Blue Heron (*Ardea herodias*)**		●	●					●		●						●				
**Green Heron (*Butorides virescens*)**									●							●		●	●	
**Killdeer (*Charadrius vociferus*)**			●	●			●	●												
**Mallard (*Anas platyrhynchos*)**	●	●	●		●	●	●	●	●		●				●	●	●	●	●	●
**Northern Rough-winged Swallow (*Stelgidopteryx serripennis*)**	●	●	●	●	●	●	●	●	●	●						●				
**Ring-billed Gull (*Larus delawarensis*)**	●	●	●	●	●	●	●	●	●	●	●	●	●	●	●	●	●	●	●	●
**Red-winged Blackbird (*Agelaius phoeniceus*)**	●		●					●	●	●						●		●	●	●
**Tree Swallow (*Tachycineta bicolor*)**							●	●												
**Wood Duck (*Aix sponsa*)**			●																	

**Fig 2:**
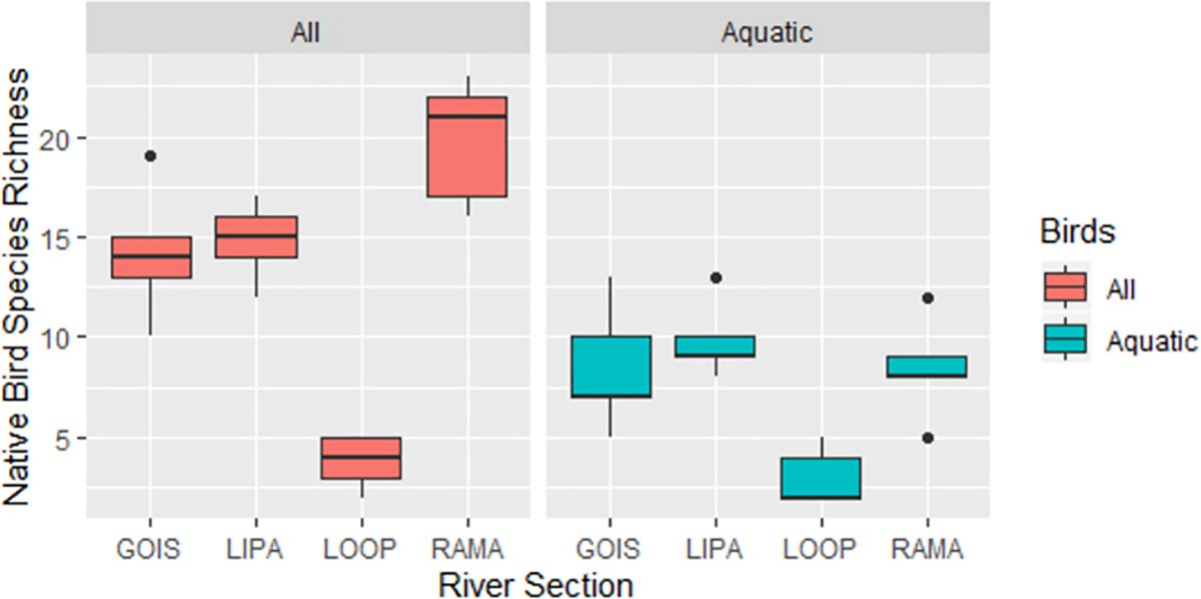
Boxplots comparing native bird species richness of all birds (left) and “aquatic” birds (right) among the four sections of the river described in the methods. The five study points of each river section are combined (n = 5 for each section). When species richness of all native birds is compared, differences between GOIS and LIPA are not statistically significant, but all other differences are significant (Wilcoxon test, p < 0.05). When only aquatic bird species richness is compared, only LOOP differs significantly from the other groups.

Species richness of all native birds was best explained by and positively correlated with canopy cover within 1 km of a point (Table 2) while species richness of aquatic birds was best explained by and negatively correlated with population density (Table 3). When species richness of all native birds is compared, differences between groups GOIS and LIPA are not statistically significant, but all other differences are significant (Wilcoxon test, p < 0.05). RAMA had the highest bird species richness and LOOP had the lowest bird species richness. When only aquatic bird species richness is compared, only LOOP differs significantly from the other groups, having the lowest aquatic bird species richness (Fig 2).

**Table 2:** AIC table for single explanatory variable linear models predicting native bird species richness, compared with a null model.

Variable	K	AICc	ΔAICc	AICc Weight	LL	Coefficient	Adjusted R^2^
**Canopy within 1 km**	3	119.51	0.00	0.95	-56.00	4.94 ± 0.96	0.57
**Canopy within 50 m**	3	125.49	5.98	0.05	-58.99	4.31 ± 1.12	0.42
**Population Density**	3	132.39	12.88	0.00	-62.44	-3.05 ± 1.33	0.18
**Null**	2	134.75	15.24	0.00	-65.02	13.15 ± 1.43	0.00
**Distance to a Major Road**	3	137.54	18.03	0.00	-65.02	0.13 ± 1.51	-0.06

Explanatory variables were standardized by subtracting the mean and dividing by the standard deviation of the sample. For each variable I give the estimate of the coefficient ± the standard error. The coefficient given for the null model is the intercept.

**Table 3:** AIC table for single explanatory variable linear models predicting aquatic bird species richness, compared with a null model.

Variable	K	AICc	ΔAICc	AICc Weight	LL	Coefficient	Adjusted R^2^
**Population Density**	3	105.40	0.00	0.69	-48.95	-1.87 ± 0.68	0.26
**Canopy within 1 km**	3	108.42	3.03	0.15	-50.46	1.46 ± 0.73	0.14
**Null**	2	109.67	4.28	0.08	-52.48	7.40 ± 0.77	0.00
**Canopy within 50 m**	3	110.93	5.53	0.04	-51.71	0.93 ± 0.78	0.02
**Distance to a Major Road**	3	111.59	6.19	0.03	-52.04	-0.71 ± 0.79	-0.01

Explanatory variables were standardized by subtracting the mean and dividing by the standard deviation of the sample. For each variable I give the estimate of the coefficient ± the standard error. The coefficient given for the null model is the intercept.

**Fig 3:**
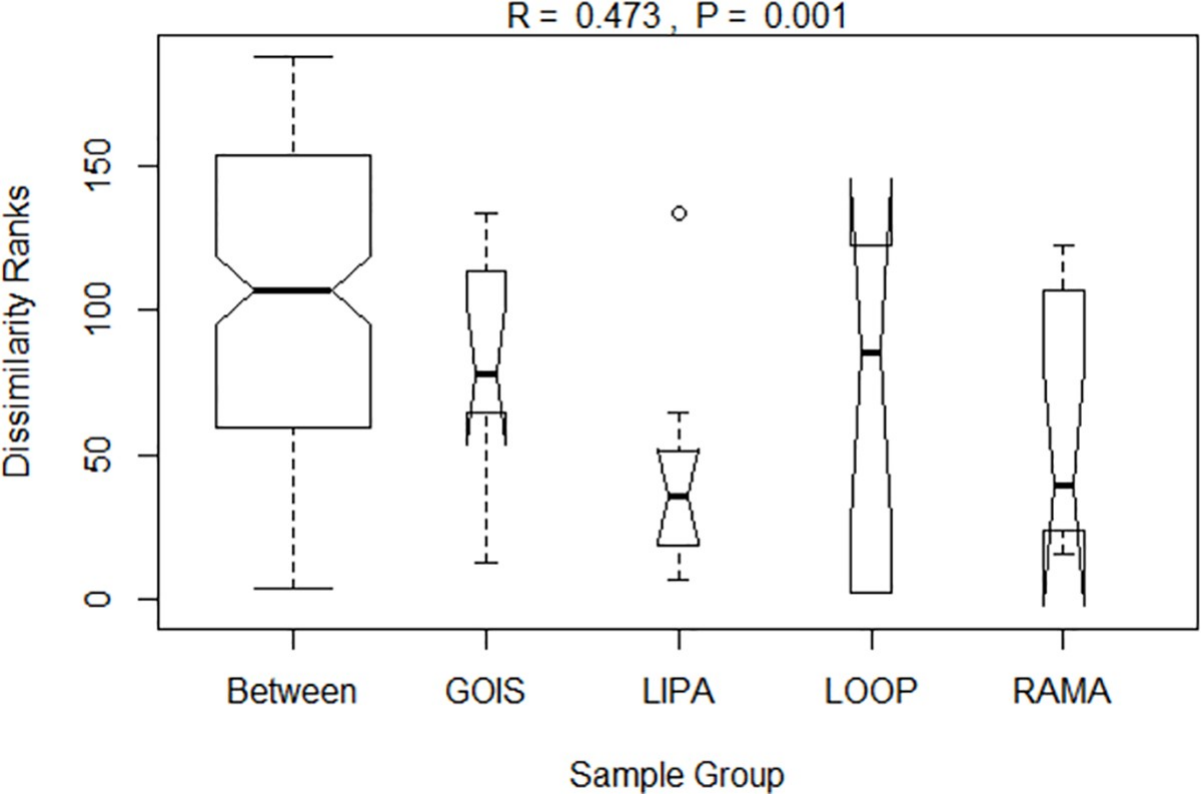
Analysis of similarity (ANOSIM) of aquatic bird communities to visualize within and between group dissimilarities. Dissimilarity ranks between and within river sections using Whittaker’s beta diversity. Communities are more similar within groups than between groups (p = 0.001), but there is still a lot of variability (high and low dissimilarity) within groups (R = 0.473). An R value close to 1 would suggest strong dissimilarity between groups, while an R value close to 0 would suggest an even distribution of high and low ranks of dissimilarities between and within groups.

**Fig 4:**
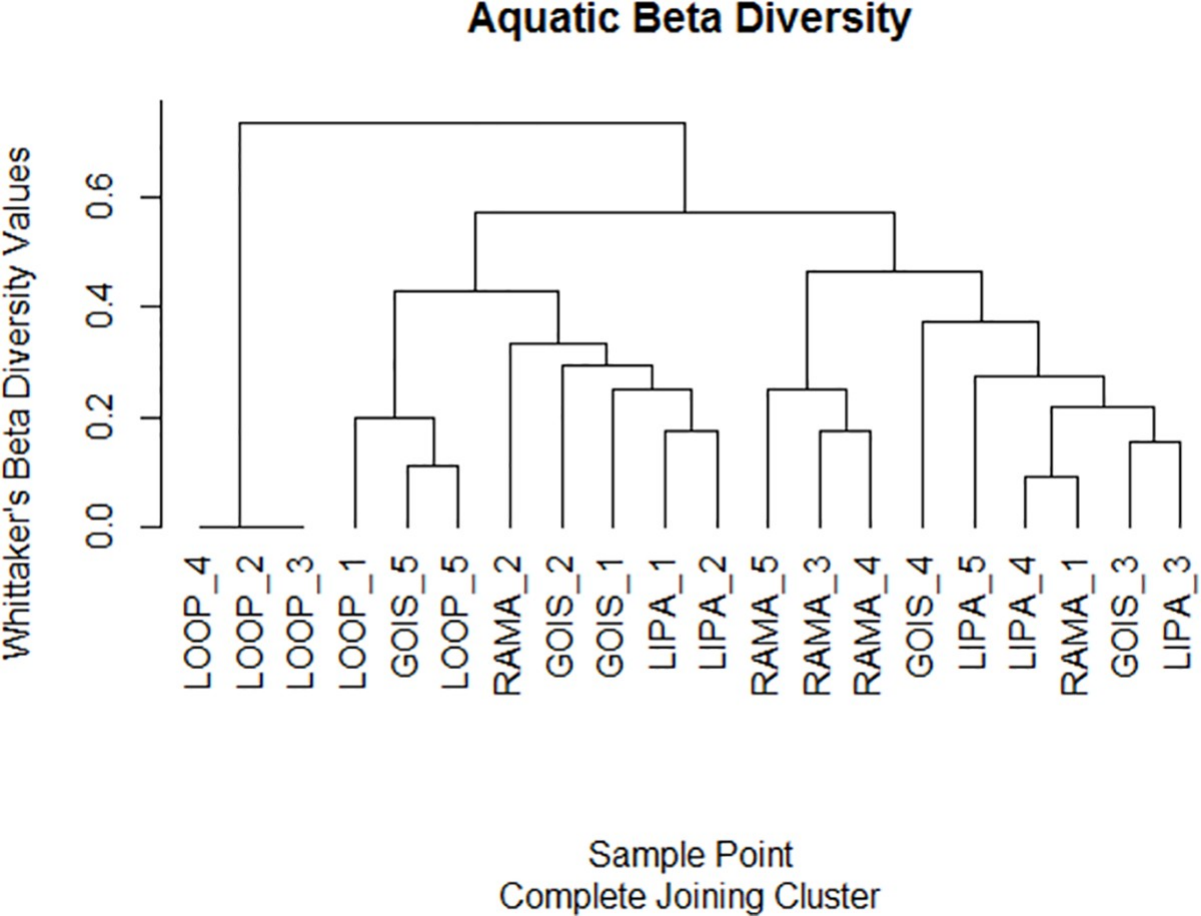
Cluster dendrogram of aquatic bird communities at the 20 study points based on Whittaker’s beta diversity metric. Communities that are closer together in the dendrogram have more similar species composition. The height of the “fork” indicates Whittaker’s beta diversity value between two study points.

Aquatic bird communities were more similar within river sections than between river sections, but there was still a lot of overlap in species composition (ANOSIM R = 0.473, P = 0.001; Fig 3). Some of the communities were more similar to those in other river sections than they were to communities in their own river sections (Fig 4). Notably, RAMA_1 was most similar to LIPA_4, in spite of the fact that RAMA_1 is located near a park with dedicated biodiversity management, and LIPA_4 is adjacent to the parking lot of a big box store.

## Discussion

The Chicago River’s north branch intersects multiple urban land uses, including residential, industrial, commercial, and recreational. The north branch also supports a diversity of birds exploiting a variety of resources and structures along the river as habitat. While total native bird species richness was highest in areas of higher canopy cover at the landscape scale, aquatic associated bird species richness was highest where human population density was lowest. Aquatic associated birds exploited neglected industrial and commercial areas even without explicit biodiversity management.

The heterogeneity in land uses and management styles probably contributed to higher beta diversity along the north branch. This result is encouraging, because urbanization can sometimes lead to biotic homogenization [21]. Much of the vegetation in the least managed river section (LIPA) is comprised of introduced species such as white mulberry (*Morus alba*), which can lead to biotic homogenization if they become ubiquitous [22]. However, if these species are tolerated in some sections and suppressed in other, more managed sections, there can still be vegetation heterogeneity at larger scales. Furthermore, allowing some sections to remain unmanaged may better accommodate the needs of bird species sensitive to human presence.

Unsurprisingly, the section of the river with the most extensive biodiversity management (RAMA) had the highest overall bird species richness. In terms of aquatic associated birds, however, its species richness and community composition were comparable to less intentionally managed river sections. Perhaps future greening efforts should have more aquatic-associated species-specific targets. Many of these targets could be met without the “park, café, and a riverwalk” model that contributes to gentrification and displacement [23]. For example, creating surfaces in the sides of the river where bank swallows (*Riparia riparia*) could nest [24] would likely be low cost and attract little attention. Provisioning of woody debris could be similarly inconspicuous. Conservation groups should pursue other “just green enough” [23] tactics such as these, particularly in areas susceptible to eco-gentrification.

There are social and conservation benefits to engaging the public and incorporating biodiversity conservation into recreation. However, in some contexts these tactics can contribute to gentrification and displacement. The narrative surrounding the section of the river near Lathrop Homes (LIPA) is that it has been “overlooked” and needs new energy [25]. Yet this study demonstrates that this section of the river supports aquatic bird diversity comparable to that of parts of the river more intentionally managed for wildlife. There is something about this part of the river, exactly as it is, that these birds are choosing. If this area is optimized for recreation, it is possible that it could become less attractive to birds.

## Conclusion

There are myriad ways to support wildlife and biodiversity in a city. Where the residents can sustain it, projects like Riverbank Neighbors Park (in RAMA) create truly special places for humans and wildlife alike. In areas that are primarily commercial, such as around Goose Island (GOIS), biodiversity-focused recreation offers valuable experiences that can be incorporated into daily life. Concentrating recreation and commerce in an area like the loop (LOOP) may not support biodiversity locally, but it may spare other parts of the river from such high levels of human activity. Where the most economically vulnerable human residents are, such as near Lathrop Homes (in LIPA), a “just green enough” approach might be best. The Chicago River is big enough for multiple management approaches to support a diversity of both wildlife and humans. Furthermore, a diversity of management approaches may lead to greater diversity at the landscape scale.
